# Epigenetic predisposition to expression of *TIMP1 *from the human inactive X chromosome

**DOI:** 10.1186/1471-2156-6-48

**Published:** 2005-09-29

**Authors:** Catherine L Anderson, Carolyn J Brown

**Affiliations:** 1 Department of Medical Genetics, University of British Columbia, 2350 Health Sciences Mall, Vancouver, BC, CANADA V6T 1Z3

## Abstract

**Background:**

X inactivation in mammals results in the transcriptional silencing of an X chromosome in females, and this inactive X acquires many of the epigenetic features of silent chromatin. However, not all genes on the inactive X are silenced, and we have examined the *TIMP1 *gene, which has variable inactivation amongst females. This has allowed us to examine the features permitting expression from the otherwise silent X by comparing inactive X chromosomes with and without *TIMP1 *expression.

**Results:**

Expression was generally correlated with euchromatic chromatin features, including DNA hypomethylation, nuclease sensitivity, acetylation of histone H3 and H4 and hypomethylation of H3 at lysines 9 and 27. Demethylation of the *TIMP1 *gene by 5-azacytidine was able to induce expression from the inactive X chromosome in somatic cell hybrids, and this expression was also accompanied by features of active chromatin. Acetylated histone H3 continued to be observed even when expression was lost in cells that naturally expressed *TIMP1*; while acetylation was lost upon *TIMP1 *silencing in cells where expression from the inactive X had been induced by demethylation. Thus ongoing acetylation of inactive X chromosomes does not seem to be simply a 'memory' of expression.

**Conclusion:**

We propose that acetylation of H3 is an epigenetic mark that predisposes to *TIMP1 *expression from the inactive X chromosome in some females.

## Background

Studies have shown considerable individual variability in the level of expression of genes (e.g. [1,2]). In general, however, humans cannot tolerate imbalances for expression of substantial numbers of genes, as demonstrated by the lethality of the majority of chromosomal aneuploidies. Aneuploidy for the sex chromosomes is better tolerated, being observed in approximately 1/500 births [3], presumably because all but one X chromosome is inactivated. X chromosome inactivation ensures the dosage equivalence of X-linked genes between females who have two X chromosomes and males who have a single X chromosome and the sex-determining Y chromosome [4]. However, more than 15% of human X-linked genes escape inactivation, being expressed from both the active and inactive X chromosome [5]. While such an escape from inactivation may maintain dosage equivalence for X-linked genes with Y homologs, the majority of human genes that escape inactivation no longer have functional Y equivalents, and thus may show relative overexpression in females (reviewed in [6]). Substantially fewer genes have been shown to escape inactivation in mice. Although this species difference in expression could reflect less extensive murine expression surveys, a reduced number of genes escaping inactivation is supported by the less drastic phenotype caused by monosomy of the X chromosome in mice (reviewed in [7]). In humans, the over or under-expression of genes that escape inactivation is a major contributor to the phenotypes associated with X chromosome aneuploidies, but may also contribute to expression differences between chromosomally normal males and females (e.g. [8]).

The study of genes that escape X inactivation can provide insight into such phenotypes, as well as contributing to our understanding of epigenetic silencing mechanisms. Inactivation occurs early in mammalian development, and the stable silencing of the X chromosome involves the acquisition of many features of heterochromatin. It is not known if escape from inactivation is a resistance to the initial silencing event, or rather reflects a high frequency of reactivation of an initially silenced gene, as data appear to support both possibilities. Many genes that escape inactivation in humans are clustered together, which may be indicative of regions that are resistant to the initial signal (e.g. [9]). However, analysis of *Smcx*, one of the few mouse genes expressed from the murine inactive X, has shown reactivation of the gene during early development [10]. Surprisingly, recent results have shown that *Smcx *has a histone modification pattern suggested to demarcate biallelically rather than monoallelically-expressed (imprinted and other X-linked) genes [11], suggesting that the gene is committed to escape inactivation prior to undergoing inactivation.

In addition to the genes that are subject to, or escape from, inactivation, there are some human genes that show heterogeneous X chromosome inactivation, being expressed from the inactive X in some females, but silenced on the inactive X in others [5,12,13]. Such genes provide an opportunity to study the same region when silent or active on an inactive X chromosome; and can thus provide insights into the features allowing expression from the inactive X chromosome. The human inactive X chromosome maintains its silent status when isolated in a mouse/human somatic cell hybrid, providing a model system to study the inactive X chromosome apart from its active counterpart. The largest survey of gene expression from the inactive X chromosome [5] analysed expression in a panel of nine inactive-X containing hybrids. That study defined heterogeneous inactivation as expression in three to six of the nine hybrids, which was observed for 60 of the 624 X-linked genes analysed. This variable expression is not restricted to the hybrid system, but has also been demonstrated in cells from females [5,12,13].

We now report the further characterization of one of these genes, the X-linked tissue inhibitor of metalloproteinases, *TIMP1*, located in Xp11.23. Our previous studies have demonstrated that *TIMP1 *is variably expressed from the inactive X in both somatic cell hybrids and human females. When *TIMP1 *is expressed from the inactive X, flanking genes (including *ARAF1 *and *ELK1 *which lie ~20 and 55 kb from *TIMP1*, respectively) are not expressed, suggesting that expression is being controlled in a gene-specific rather than regional fashion [12]. Quantitative RNase protection assays showed substantial variability in expression levels from the active X, precluding using expression levels to determine inactive X expression in females. Further studies in hybrids demonstrated that *TIMP1*-expressing clones were unstable and that methylation does not appear to be the principle controlling feature allowing variable expression of *TIMP1 *from the inactive X chromosome [14]. In this study we report the analysis of other features characteristic of an inactive X to determine which features might predispose *TIMP1 *to expression from the inactive X chromosome in a subset of females.

The inactive X acquires many of the general features of heterochromatin (reviewed in [15]), and we have now examined replication timing, nuclease sensitivity, and histone modifications for *TIMP1*. Late replication of the inactive X at the chromosome level is observed after Giemsa staining following bromodeoxyuridine incorporation [16]. Regions such as the distal and proximal short arm that contain a large proportion of genes that escape inactivation are not delayed in their replication, supporting a regional basis to escape from inactivation. Replication of individual X-linked genes has been analysed by fluorescent in situ hybridization (FISH) or amplification of BuDR incorporated DNA after flow cytometry. Although correspondence is not complete between different techniques [17], such methods have generally shown that genes that escape inactivation are early replicating (reviewed in [18]). DNase sensitivity is a general feature of active chromatin, and promoters of genes subject to inactivation have been seen to be less available for digestion by nucleases (*e.g. *[19]). Modifications to the histones associated with the inactive X are reflective of both general heterochromatic changes and ones specific to the facultative heterochromatin of the inactive X. Using antibodies to acetylated histones the inactive X chromosome stains very palely [20], while antibodies to histone H3 methylated at lysine (K) 9 (H3mK9) are generally associated with heterochromatin and those to methylated lysine 27 (H3mK27) specifically mark the inactive X chromosome [21,22]. Chromatin immunoprecipitation (ChIP) has revealed that genes subject to inactivation show limited acetylation and elevated histone methylation (H3mK9/27) at their promoters relative to genes escaping inactivation [23,24].

## Results

### Replication timing of *TIMP1 *in human cells

**Figure 1:**
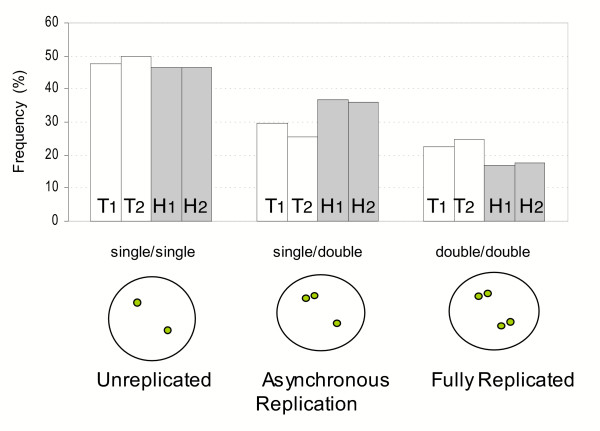
Replication asynchrony for the *TIMP1 *(T – white bars) and *HPRT *(H – grey bars) genes assessed by FISH. The bars show the frequency of nuclei exhibiting unreplicated (single/single); asynchronously replicated (single/double) and completely replicated (double/double) signals as shown in the schematic below. The *TIMP1 *and *HPRT *probes were individually hybridized to interphase nuclei of the same preparations. Two human female lymphoblast cell lines were examined for the approximate degree of replication asynchrony, one that inactivated *TIMP1 *(1 – GM07059) and one that expressed *TIMP1 *from the Xi (2 – HSC593). For *TIMP1*, 313 cells were counted from 3 separate cell harvests for cell line 1 (T1) and 217 cells for cell line 2 (T2) from two separate harvests. 144 cells were examined for *HPRT *from 2 slides of one harvest for cell line 1 (H1) and 97 cells from a single harvest for cell line 2 (H2). Between different cell harvests of the same cell line the maximal difference in frequencies of nuclei in each of the three replication classes was 3%.

**Figure 2:**
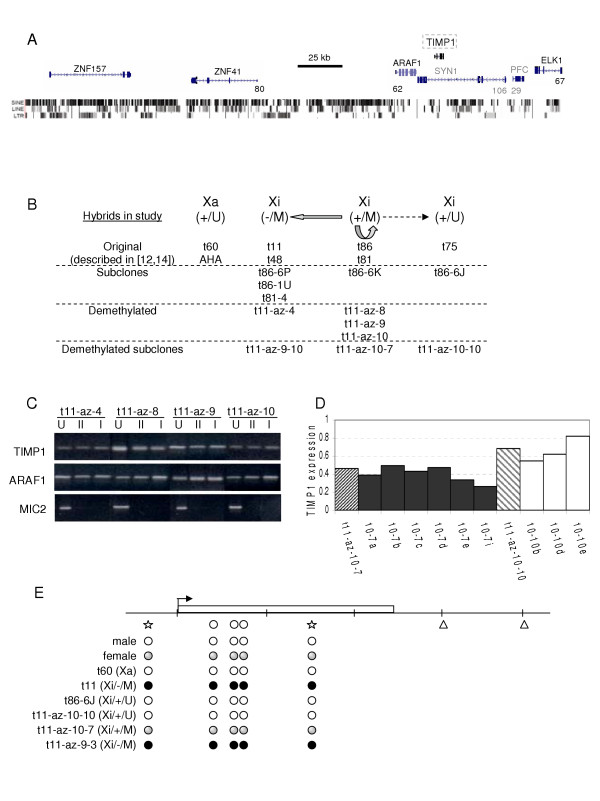
Methylation of *TIMP1*. A. The genes in the region surrounding *TIMP1 *are shown with the gene name written above the line diagram for the gene (with vertical lines representing exons and small arrows showing transcriptional orientation). The number listed on the line below at the 5' end of the genes indicates the CpG island density. The *TIMP1 *and *ZNF147 *genes do not have enough CpG sites to qualify as a CpG island so there is no number listed. The *SYN1*, and *PFC *genes are listed in grey because these genes (which are expressed in a tissue-specific manner) were not examined in this study. The presence of repeat elements (SINE, LINE and LTR) is indicated by the vertical lines below the genes. This figure is based on data generated by the UCSC browser  hg17 of NCBI Build 35. B. Somatic cell hybrids analysed for *TIMP1 *activity. In addition to the stable active X chromosomes that express *TIMP1 *and are unmethylated at the 5' end of the gene (Xa/+/U) there are several categories of inactive X chromosomes. Most inactive X chromosomes previously analysed do not express *TIMP1 *and are methylated (Xi/-/M). Other inactive X chromosomes express *TIMP1 *and are unmethylated (Xi/+/U), while an intermediary class of hybrids showed both DNA methylation and lower expression levels (Xi/+/M). Subcloning of these latter cells showed that they were unstable, giving rise to additional methylated expressing clones as well as methylated silent clones and expressing unmethylated clones. The arrows show approximate proportion of cells of each class derived from subcloning. Subclones further characterized are listed below, as are clones derived by 5-azacytidine treatment and their subclones (see Tables 1 and 2). C. DNA methylation of clones from four of the demethylated clones listed in Table 1. DNA from each clone was digested with *Eco*RI alone (U), *Eco*RI plus *Hpa*II (II) or *Eco*RI plus *Hha*I (I). Primers for the 5' end of *TIMP1 *and *ARAF1 *that flank *Hpa*II or *Hha*I methylation-sensitive restriction enzyme sites were used to assess methylation, while amplification of *MIC2*, which is unmethylated on both active and inactive X chromosomes, served as a control for complete digestion with the methylation-sensitive enzymes. D. Comparison of expression levels in subclones of two 'sibling' subclones of t11-az-10 differing in methylation states. The t11-az-10-7 and t11-az-10-10 clones are striped, with their subclones shown to their right. Methylation (dark fill) was observed for t11-az-10-7 and its subclones while t11-az-10-10 and its subclones were unmethylated (unfilled). All subclones continued to express both *TIMP1 *and *ARAF1 *as assayed by RT-PCR. Despite the relative stability of the methylated *TIMP1*+ culture, the *TIMP1 *expression level was significantly lower in the methylated cultures (p < 0.01). E. Methylation analysis by bisulphite treatment. The 5' end of the *TIMP1 *gene was sequenced after bisulfite conversion, which changes unmethylated Cs to Us but leaves methylated Cs unchanged. Therefore, the presence of a C indicates that the CpG was methylated. The following CpG sites were analyzed: *Hha*I sites (stars) at -3 and +31; and three other sites not analyzed by methylation-sensitive enzymes (circles) at +11, +17, +20. The *Hpa*II sites (triangles) at +61 and +81 were used in methylation-sensitive assays but were not reliably analysed by bisulphite sequencing. The open circles indicate unmethylated CpGs whereas the filled circles represent methylated CpGs. The shaded circles designate that both converted and unconverted bases were seen after sequencing, indicating that both methylated and unmethylated CpGs were present. Cell lines are listed, the male cells were GM7057 and the female cells were GM7059. t11-az-9-3 is a *TIMP1*^- ^subclone of t11-az-9.

To evaluate the influence of replication timing upon expression of *TIMP1*, cell lines from a female with and a female without *TIMP1 *expression from the inactive X [12] were analysed by DNA FISH using probes for the *TIMP1 *and *HPRT *loci. As shown in the schematic of Figure 1, cells that have not replicated either X chromosome will show two single signals, and almost one half of the cells were of this type. Of the cells with at least one double signal, indicating replication of the locus, considerable asynchrony was observed for the *HPRT *locus, as 37% of all cells analysed showed single-double (SD) signals and 17% showed the double-double (DD) signals. The *TIMP1 *locus generally showed lower asynchrony of replication than *HPRT *(28% of cells were S/D vs 37% for *HPRT*, P < 0.0001). The cell line with *TIMP1 *expression from the inactive X (cell line 2 – HSC593) showed a small trend towards having a lower percentage of cells that replicated asynchronously than the GM07059 cell line which does not have *TIMP1 *expression from the inactive X (25% vs 29%); however this difference was not significant (P = 0.17). The observed shift in replication timing, while not statistically significant, may be a contributing factor in the expression of *TIMP1 *from the inactive X. However, it is likely that additional changes are also involved, as *TIMP1 *is within 20 kb of the *ARAF1 *gene and 55 kb from *ELK1 *(see Figure 2A), and thus at least one of these genes is likely to share a replication domain with *TIMP1 *despite remaining silent when *TIMP1 *is expressed [12].

### DNA methylation of *TIMP1 *in somatic cell hybrids

Analysis of alterations to the inactive X chromosome is complicated by the presence of the active X chromosome in female cells, so we have analysed features of inactive X chromosomes isolated in mouse/human somatic cell hybrids. We have previously described the characterization of DNA methylation status and expression levels in a number of these hybrids [14] and Figure 2B presents an outline of the hybrids analysed in this study. In addition to hybrids retaining the active X chromosome (t60-12 (t60) and AHA11aB1 (AHA)), in which *TIMP1 *was unmethylated and expressed, three classes of inactive X-containing hybrids were previously described. Those that were methylated and did not have *TIMP1 *expression (Xi/-/M – t11-4Aaz-5 (t11) and t48-1a-1Daz4a (t48)) or expressed *TIMP1 *and were unmethylated (Xi/+/U – t75-2maz34-1a (t75)) were stable, with no gain or loss of expression. In contrast, the two hybrids that expressed *TIMP1 *but were methylated at the *TIMP1 *promoter (Xi/+/M – t86-B1maz1b-3a (t86) and t81-az1D (t81)) demonstrated instability as subclones could be either methylated and expressing (Xi/+/M) or methylated and silent (Xi/-/M) as well as occasionally unmethylated and expressing (Xi/+/U). Consistently the Xi/-/M and Xi/+/U subclones tended to be stable while Xi/+/M subclones were unstable. As the methylated-expressing clones (Xi/+/M) are unstable and give rise to a mixed population of methylated silent and unmethylated expressing clones, these clones do not indicate if loss of methylation follows, or predisposes to, expression.

To further test the role of methylation in regulating expression in the *TIMP1 *region, an inactive X-containing hybrid that did not express *TIMP1 *(t11) was treated with 5-azacytidine, which is known to induce reactivation of X-linked genes in somatic cell hybrids. As there is not a selectable marker for *TIMP1 *reactivation, clones were initially selected for *HPRT *reactivation to ensure that the clones had been demethylated and potentially increase the frequency of reactivation, as co-reactivation of different X-linked genes has been observed after 5-azacytidine treatment [25]. 15 *HPRT*+ clones were selected in HAT media, and examined by RT-PCR for *TIMP1 *as well as flanking gene expression (Table 1). After three passages, six clones showed expression of *TIMP1*. A number of flanking genes were also observed to reactivate with 5-azacytidine treatment; with one clone expressing *ARAF1*, five expressing *ELK1*, three expressing *ZNF41 *and eight expressing *ZNF157*. The single *ARAF1*-expressing clone, and all five *ELK1*-expressing clones, were observed in *TIMP1*+ clones, and all three *ZNF41 *positive clones were *ZNF157 *positive. The *TIMP1 *and *ZNF157 *genes lack a CpG island (see Figure 2A), and *TIMP1 *and *ZNF157 *showed the highest (6/15 and 8/15) reactivation frequencies after 5-azacytidine treatment. However, *ARAF1 *showed the lowest reactivation frequency (1/15) despite having a smaller CpG island than either *ELK1 *or *ZNF41*, so reactivation frequency was not simply a reflection of CpG density.

**Table 1:** Expression of *TIMP1 *and surrounding genes following 5-azacytidine induced reactivation of *HPRT*

Clone *	*TIMP1*	*ARAF1*	*ELK1*	*ZNF41*	*ZNF157*
**t11-az-4**	**-**	**-**	**-**	**-**	**+**
t11-az-5	-	-	-	-	+
t11-az-6	+	-	+	-	-
t11-az-7	+	-	+	-	-
**t11-az-8**	**+**	**-**	**+**	**-**	**-**
**t11-az-9**	**+**	**-**	**+**	**-**	**+**
**t11-az-10**	**+**	**+**	**-**	**+**	**+**
t11-az-11	-	-	-	+	+
t11-az-14	-	-	-	-	+
t11-az-16	-	-	-	-	+
t11-az-17	-	-	-	-	-
t11-az-18	-	-	-	+	+
t11-az-19	+	-	+	-	-
t11-az-20	-	-	-	-	-
t11-az-21	-	-	-	-	-

*The clones in bold were ones that were further subcloned to assess stability (see Table 2).

Despite 5-azacytidine treatment and subsequent expression in some clones, DNA methylation continued to be observed at 5' end of *ARAF1 *and *TIMP1 *in these clones, as shown in Figure 2C. Given our previous results demonstrating instability of expression in the presence of methylation, four clones were subcloned after 7 weeks of culture. Analysis of expression and DNA methylation for *TIMP1 *(Table 2) showed that, as expected, all subclones of a non-expressing clone (t11-az-4) remained silent. For both t11-az-8 and 9, the majority of clones lacked *TIMP1 *expression. Only two weakly positive clones were identified for each line, and even these subclones lost expression by 12 weeks in culture. Three subclasses of hybrids were observed for the t11-az-10 subclones, reminiscent of the situation seen in hybrids from females who spontaneously express *TIMP1 *from the inactive X. t11-az-10 was the only reactivated clone that expressed *ARAF1*, and nine of the 14 *TIMP1*-expressing subclones expressed *ARAF1*. The single subclone that was unmethylated at *TIMP1 *(t11-az-10-10) expressed *TIMP1*, and also expressed *ARAF1*, which was also unmethylated. t11-az-10-7 was methylated yet expressing for both *TIMP1 *and *ARAF1*. These clones (t11-az-10-10 and t11-az10-7) were further subcloned. The *TIMP1 *expression level of the subclones was determined by RNase protection. The t11-az-10-7 subclones retained DNA methylation, and expressed a lower level of *TIMP1 *than the unmethylated t11-az-10-10 subclones (Figure 2D).

**Table 2:** Stability of methylation and expression of *TIMP1 *in subclones of four 5-azacytidine induced *HPRT *reactivants

Subclones	t11-az-4 (-/M)	t11-az-8 (+/M)^a^	t11-az-9 (+/M)^a^	t11-az-10 (+/M)
TIMP-, methylated (-/M)	10	10	15	3
TIMP+, Methylated (+/M)	0	2^a^	2^a^	13
TIMP+, Unmethylated (+/U)	0	0	0	1

^a^Expression in these branches was initially weak and became silent after time in culture. *ELK1 *expression was initially retained in one of the t11-az-9 *TIMP1*+ clones but was not analyzed after *TIMP1 *expression was lost. The expression of *ZNF41/157 *was not examined in these subclones.

DNA methylation analysis was routinely based on methylation-sensitive restriction enzyme digestion followed by PCR that examined 4 sites, 3 of which were over 50 bp downstream of the transcription start site (Figure 2E). To examine methylation of additional CpG dinucleotides closer to the promoter, methylation analysis by bisulfite modification followed by sequencing of PCR products was performed. Three additional CpGs near the *TIMP1 *transcription start yielded the same methylation pattern as had been observed at the restriction enzyme sites. Direct sequencing of the PCR product was performed to allow the identification of heterogeneous populations. This approach is complementary to the methylation-sensitive restriction enzyme approach, which gave a positive signal (amplification) for low levels of methylated DNA. The high background corresponding to the unconverted (methylated) Cs, presumably due to their under-representation in the sequence, prohibits identification of low levels of methylated bases by direct sequencing. However, no background of the converted base (A) was present and thus this technique is sensitive to the presence of low levels of unmethylated DNA. If the peak corresponding to the converted base (A) was demonstrably higher than the unconverted (G), the site was called unmethylated (white circles in Figure 2E). Completely methylated sites (as seen for the inactive X hybrid) are indicated by filled in black circles. Sequencing also yielded a mix of unmethylated (converted) and methylated (unconverted) sites. This was observed in a female cell line, where the methylated (unconverted – G) peak height was greater than or equal to the unmethylated (converted – A) peak height. Such a mix, demarcated by the grey circles, was also observed for the t11-az-10-7 cells, although in this case the unmethylated peak was consistently lower than the methylated peak.

### DNase sensitivity of *TIMP1 *in somatic cell hybrids

**Figure 3:**
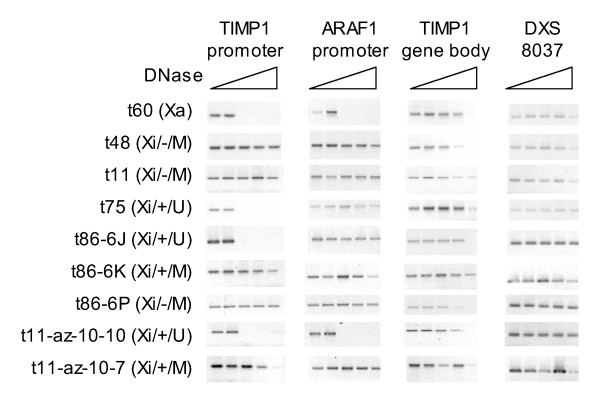
Nuclease sensitivity of *TIMP1*. Nuclei from various cell lines (as indicated on the left) were treated with increasing amounts of DNase I (0, 0.1, 0.25, 0.5, 1.0 units) as represented by the increasing breadth of the triangle. The DXS8037 primers flank a non-coding region and were used to check that equal digestion and PCR amplification occurred across all cell lines. The cell line designations indicate X activity, *TIMP1 *expression status, and methylation status as shown in Figure 2B.

Nuclease sensitivity of the *TIMP1 *promoter, the promoter of the nearby *ARAF1 *gene, as well as the *TIMP1 *gene body and the anonymous DNA marker DXS8037 was assessed in a series of these clones (Figure 3). The promoters of *TIMP1 *and *ARAF1 *were sensitive to nuclease on the active, but not the inactive X chromosome, while the gene body or intergenic regions were generally resistant on both active and inactive X chromosomes. The *TIMP1 *promoter was also sensitive to digestion in the unmethylated expressing (Xi/+/U) *TIMP1 *clones, while it was insensitive in both the *TIMP1*-expressing, and silent methylated clones (Xi/+/M and Xi/-/M). A similar effect was observed for the demethylated clones, with both *TIMP1 *and *ARAF1*, being expressed in both the t11-az-10-10 and t11-az-10-7 clones, but only sensitive to nuclease digestion in the t11-az-10-10 clone, where they were unmethylated.

### Histone modifications

**Figure 4:**
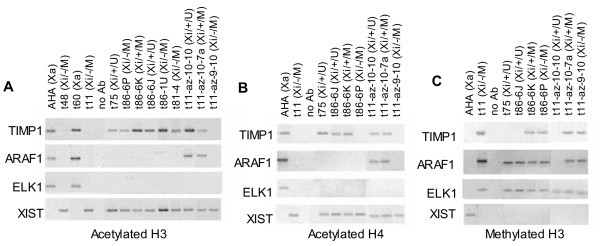
ChIP analysis of *TIMP1 *expressing or non-expressing hybrid clones. PCR products were amplified from DNA at various X-linked gene promoters after ChIP using antibodies to the modifications listed below the panels for the cell lines listed across the top of each panel (see Figure 2B for derivation of lines). t11-az-10-7a is a subclone of t11-az-10-7 (see Figure 2D). The DNA template for 'no Ab' lanes was prepared following the ChIP procedure without an antibody. A. PCR amplification products after ChIP with acetylated histone H3. Similar analysis to A, using antibody to acetylated histone H4 (panel B), or methylated histone H3 (C).

A growing number of specific modifications to the histone tails have been associated with both constitutive and facultative heterochromatin. To assess the potential role of such modifications in the escape from inactivation of *TIMP1*, we analysed the hybrids by ChIP with antibodies to acetylated histone H3 (antibody recognizes acetylated K9 and 14), acetylated histone H4 (antibody recognizes acetylated K 5, 8, 12, and 16) and methylated histone H3 (antibody to di-methyl K9, although the antibody will cross-react with tri-methyl K27 see: ). The promoter regions of *TIMP1, ARAF1, ELK1 *and the *XIST *gene (the latter is expressed solely from the inactive X chromosome) were analysed (shown in Figure 4). Additional analyses of the promoters of the *PGK1 *gene that is expressed only from the active X chromosome, and the *ZFX *gene that is expressed from both active and inactive X chromosomes, gave the anticipated results for active and inactive X chromosomes (data not shown). Antibody to acetylated H3 immunoprecipitated the *TIMP1*, *ARAF1 *and *ELK1 *promoters for the active, but not inactive X-containing hybrids, while the reverse was observed for the *XIST *promoter. *ELK1 *was not expressed in the inactive X hybrids examined, and was not immunoprecipitated. However for *TIMP1 *and *ARAF1 *association with acetylated histone H3 was observed for all expressing clones whether on the active or inactive X chromosome. In addition, immunoprecipitation of the *TIMP1 *promoter was observed for Xi/-/M clones (t86-6P/t86-1U and t81-4). Immunoprecipitation was completely concordant with expression for the acetylated histone H4 antibody at all of the genes examined. Methylation of H3 at K9 corresponds to silent chromatin, and thus, as anticipated, results were generally opposite to those seen for acetylation. Immunoprecipitation was observed when the gene was silent – i.e. *XIST *on the active X and *TIMP1*, *ARAF1 *and *ELK1 *on the inactive X. However, in all cases where there was expression in the presence of ongoing promoter methylation (for both *ARAF1 *and *TIMP1*) the promoter was immunoprecipitated by the methylated H3 antibody. Thus we detect a distinctive pattern of histone acetylation and methylation that does not correspond simply with the expression or DNA methylation status of the *TIMP1 *gene.

## Discussion

The facultative heterochromatin of the inactive X chromosome is a fascinating system to study the epigenetic modifications associated with silent chromatin as both an active and inactive version of most X-linked genes exist in female cells. However, not all genes on the 'inactive' X chromosome are subject to silencing [26], and these genes that escape inactivation must somehow maintain an active state on an otherwise silent chromosome. *TIMP1 *is subject to inactivation in many females, and we now show that when subject to inactivation the anticipated features of inactive chromatin are assembled – promoter DNA hypermethylation, nuclease insensitivity and histone methylation and hypoacetylation. In other females, however, we have previously demonstrated that *TIMP1 *continues to be expressed from the inactive X chromosome, and in this study we have exploited such chromosomes to analyze the epigenetic features that are associated with expression from the inactive X. By comparing expression of *TIMP1 *that occurs naturally with that induced by the demethylating agent 5-azacytidine we are also able to propose which feature may predispose to expression of *TIMP1 *in otherwise silent chromatin. We observed that expression of *TIMP1 *was associated with an active chromatin structure, despite the presence of the gene on the inactive X chromosome, except in three situations.

First, in female cell lines with or without *TIMP1 *expression from the inactive X there was a very similar extent of replication asynchrony, suggesting that the expression of *TIMP1 *was ocurring from a late-replicating region of the X chromosome. This is not surprising, as the genes flanking *TIMP1*, which are not variable in their inactivation, are located within 55 kb, so it is likely that at least one of *ARAF1 *or *ELK1 *shares a replication origin with *TIMP1*. We assessed replication timing by FISH, and detected a frequency of replication asynchrony for the *HPRT *locus in close agreement with the frequencies previously reported for this gene [27]. It has been suggested that the 'double dot' versus 'single dot' pattern may reflect chromatid association in addition or instead of replication asynchrony [17]. Regardless of the underlying cause, the results were highly reproducible and consistently showed less asynchrony for the *TIMP1 *locus relative to the *HPRT *locus, although still in the range that is generally considered asynchronous [28]. This reduced asynchrony at *TIMP1 *could reflect, or contribute to, weaker epigenetic silencing being established in this region of the X chromosome. There was a slight, but not statistically significant, trend for the female showing expression of *TIMP1 *from the inactive X chromosome to show even less replication asynchrony for *TIMP1*, however it seems unlikely that such a small difference could contribute substantially to *TIMP1 *expression from the inactive X, and thus other factors must be involved in permitting the expression of *TIMP1 *from the inactive X.

**Figure 5:**
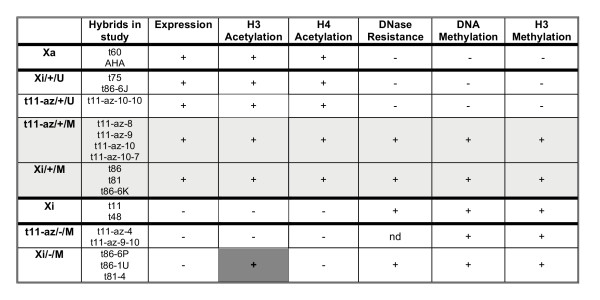
Summary of chromatin features observed in somatic cell hybrid clones for the *TIMP1 *gene. Most subclones and demethylated clones follow the patterns seen for the active and inactive X hybrids that are outlined in bold. A positive (+) designates the presence of the feature, while a negative (-) depicts the absence of the feature, while ND means that the feature has not been examined in that class of clones. The assays used are PCR-based and would detect a small population of cells. DNase sensitivity is listed as the inverse – DNase resistance – as it is the presence of undigested (resistant) DNA that will yield a 'positive' PCR signal. In each category of clones not all clones have been examined for all features. For the clones shaded in grey the results are an amalgamation of results anticipated from an active and inactive X, and we suggest these clones represent a mixed population. This suggestion is generally supported by sub-cloning experiments (see text for discussion). The ongoing H3 acetylation of the Xi/-/M hybrids (highlited in darker grey) cannot be attributed to heterogeneous cell populations, and since it is not seen for demethylated hybrids that have lost *TIMP1 *expression (t11-az/-/M) we suggest that this modification reflects a predisposing feature of inactive X chromosomes that express *TIMP1*.

The other two situations in which expression and active chromatin features were not concordant were found in the somatic cell hybrids, and these results are summarized in Figure 5. As shown in the grey box, DNA methylation and additional features of silent chromatin were detected along with expression in the Xi/+/M (t86, t81) cells. We attribute this to heterogeneity in the population of cells, as subcloning yielded both methylated/silent and unmethylated/expressing clones. In addition to DNA methylation, these clones were observed to be insensitive to DNase and have methylated H3K9 residues near the *TIMP1 *promoter. As the assays for these features relied on PCR, a positive signal could be obtained from a subpopulation of cells with a silent chromatin structure, while another population of cells could be positive for expression and active chromatin modifications (histone acetylation). We also suggest that heterogeneity accounts for expression in the presence of DNA methylation in several of the demethylated clones, including the t11-az-10-7 clone. However, unlike the mixed population of subclones obtained with all other Xi/+/M cells, eight of eight subclones of t11-az-10-7 were methylated and expressing, and six of these subclones examined by RPA showed a consistently reduced level of expression relative to their unmethylated counterparts. While it was surprising that subcloning did not isolate distinct subpopulations, the presence of an unmethylated subpopulation was detectable by bisulphite sequencing. Thus heterogeneity again seems the most likely explanation for DNA methylation, DNase insensitivity and histone H3K9 methylation in these expressing cells. However, without single cell assays it is not possible to rule out that there are methylated cells that express *TIMP1 *at reduced levels and show additional features of silent chromatin. These two classes of clones were the only exceptions to concordance between nuclease sensitivity and gene expression at the promoters of the *TIMP1 *and *ARAF1 *genes; and previous studies of the nucleosomal organization of the *HPRT1 *gene promoter have shown that methylation does not directly affect the differential positioning of nucleosomes on active and inactive X-linked promoters [19]. Thus we believe that the methylated/expressing/nuclease insensitive clones reflect the presence of a subpopulation of silent cells. This heterogeneity demonstrates the unstable nature of silencing for *TIMP1 *in these cells.

The third exception is the most interesting, and is highlighted in Figure 5 with a dark grey fill. Acetylation of histones is generally seen for active genes, and acetylated histone H4 showed complete concordance with expression for all genes. However, the Xi/-/M clones (t86-6P and 1 U as well as t81-4) showed ongoing acetylation at H3 despite having lost *TIMP1 *expression from the inactive X chromosome. The Xi/-/M subclones were derived from two different unstable, naturally expressing inactive X chromosomes (in t86 and t81), however expression of *TIMP1 *in these hybrids was now stably silenced, and thus this result was not a reflection of a mixed population of cells. Furthermore, acetylation cannot simply reflect a failure of this region to reset chromatin structure, as the demethylated t11-az-9-10 hybrid which had lost expression had also lost acetylation. Methylation of H3 K9 was observed in these silent clones, so the Xi/-/M cells, despite being a homogeneous population, appear to show both methylation and acetylation of histone H3. This may reflect the lack of specificity of the antibodies used for these experiments, as the acetylated H3 antibody recognizes acetylation at residues 9 and 14, while the antibody to methylated K9 can cross-react with methylation of K27. Thus immunoprecipitation by both antibodies may reflect modification of specific lysines. It is also possible that only a particular set of nucleosomes show the acetylation mark, and as the sonicated fragments immunoprecipitated in the assay averaged ~600 bp, modifications on several different nucleosomes flanking the primers used could result in immunoprecipitation. Regardless of the specific site of modification, acetylation of histone H3 was the one feature that was consistently associated with X chromosomes that were naturally predisposed to expression from the inactive X chromosome, regardless of expression status. Thus we propose that histone H3 acetylation differs at the *TIMP1 *genes in females, predisposing some females to expression from the inactive X. Unfortunately this hypothesis is difficult to test, as females generally show acetylation at *TIMP1*, due to the presence of the active X chromosome. It would be necessary to study clonal populations of cells from a female with a polymorphism close enough to the promoter to be analysed by ChIP, and currently no such polymorphisms are known. Our previous work did not show an association between expression of *TIMP1 *and a downstream polymorphism in exon 5 [12].

A different predisposing epigenetic mark for expression from the inactive X has previously been reported in mice. Methylation of H3 at K4 was restricted to the promoter in undifferentiated embryonic stem cells for genes that would subsequently be expressed monoallelically (*i.e. *genes subject to X inactivation or imprinting), while autosomal genes or genes that escape inactivation had H3 K4 methylation in the gene body as well as the promoter [11]. Such methylation was observed in the body of the *Smcx *gene which is initially silenced, and then reactivates, reminiscent of the H3 acetylation mark and instability of silencing that we observe for *TIMP1*. While many genes escape inactivation in humans, *TIMP1 *provides us with a unique opportunity to examine the gene in its expressed and silent state in hybrid somatic cells. Thus, it will be interesting to study additional chromatin modifications for *TIMP1*, including H3 K4 methylation, as well as additional modifications recently associated with the inactive X chromosome such as H4 K20 methylation [29] or H2A ubiquitinylation [30,31]. Even more interesting would be to determine if the changes seen are observed for additional X-linked genes that are variable in their inactivation status, if suitable model systems could be developed.

Previous examinations of genes that escape inactivation have shown an active chromatin structure (reviewed in [7]), and recent results further demonstrate that epigenetic modifications seem to be heterogeneous in their distribution along the X [32]. These 'flavours' of inactive X chromatin may correspond to clusters of genes that escape inactivation. While genes that escape inactivation tend to be clustered in blocks and enriched on the short arm of the X chromosome, genes such as *TIMP1 *that are variable in their inactivation are more evenly distributed along the X chromosome ([5], reviewed in [6]), and thus may reflect different mechanisms leading to expression from the inactive X. The CTCF boundary factor has recently been shown to be found between the domain of escape and that of inactivation [33]. It is not known whether such boundaries flank the variably inactivated genes. The five clones that showed reactivation of *ELK1 *were all *TIMP1*+, suggesting a co-ordinate reactivation between the two genes. Only one *ARAF1 *reactivant was identified, and this clone was also *TIMP1+*. However, this co-ordinate reactivation of *TIMP1 *with flanking gene(s) is different from the *TIMP1*-specific expression seen for the inactive X naturally. The *ZNF157/41 *genes did not seem to show co-ordinate reactivation with the *TIMP1 *region, however all three *ZNF41 *positive clones were *ZNF157 *positive, which suggests they may be in a separately controlled domain.

While association with a CpG island does not differ between genes subject to or escaping from inactivation, genes heterogenous in their inactivation are more likely to lack an island [5], consistent with the *TIMP1 *promoter being the least CpG dense of the region. Although *TIMP1 *lacks a CpG island, our analysis suggests that methylation of the few CpGs present near the promoter generally correlates well with silencing. Demethylation resulted in at least transient expression of all genes examined, confirming the importance of DNA methylation in stable maintenance of X chromosome silencing. Examination of the *ELK1, TIMP1 *and *ARAF1 *promoters after demethylation showed the presence of DNA methylation, even when the clones were expressing the genes. The majority of these clones subsequently resilenced these genes, as has been observed for other genes following demethylation [34], perhaps reflecting only partial initial demethylation or the spread of silencing from adjacent silent regions that retained DNA methylation or other epigenetic marks of silencing.

It has been proposed that the evolutionarily recent addition of the X short arm may predispose genes located there to escape from silencing [35]. Interestingly, *TIMP1 *is very close to the evolutionary breakpoint between the region added to the X after marsupial divergence. *ARAF1 *is found on the marsupial X while *TIMP1 *and its surrounding gene *SYN1 *are autosomal in marsupials, suggesting that they are part of the eutherian addition to the human X [36]. Since genes closely flanking *TIMP1 *are normally subject to X inactivation, even when *TIMP1 *is expressed, evolutionary history alone cannot explain the escape from inactivation, although it may result in a different genomic context that contributes to expression from the inactive X. However, inspection of genomic features in the region surrounding *TIMP1 *does not show a substantial difference in the frequency of repetitive elements near *TIMP1 *relative to the flanking *ARAF1 *and *ELK1 *genes (Figure 2A).

## Conclusion

Several factors, including reduced replication asynchrony, lower CpG density, and more recent evolutionary addition to the X, may contribute to less stringently controlled inactivation for *TIMP1*. However, we propose that there is a difference between females for a feature unique to *TIMP1 *that predisposes some females to expression of the gene. This mark appears to be at least reflected in a difference in acetylation of histone H3 on the inactive X in females predisposed to expression of *TIMP1 *from the inactive X chromosome. While histone acetylation appears to be a predisposing mark for expression of *TIMP1*, there may be a different hierarchy of epigenetic modifications permitting expression of other genes from the inactive X. Elucidation of these mechanisms is important not only as a model for epigenetic gene regulation but because genes that escape inactivation contribute to the phenotype of X chromosome anueploidies, and may also result in differential male/female expression levels and disease susceptibilities.

## Methods

### Cell culture

Lymphoblast cell lines were grown in RPMI 1640 media (Stem Cell Technologies) supplemented with 15% fetal calf serum (Cansera), L-glutamine (Invitrogen) and penicillin/streptomycin (Invitrogen). Cells were harvested 12–26 hours after addition of fresh media by centrifugation. The human/rodent somatic cell hybrids were grown in alpha minimal essential media (Invitrogen) supplemented with 7.5% fetal calf serum, L-glutamine, penicillin/streptomycin, and non-essential amino acids (Invitrogen) to sub-confluence before harvesting with trypsin-EDTA (0.25%). Cell lines have been previously described [14]. To generate single cell clones, the hybrid cultures were plated to a final concentration of 3 to 17 cells/60 mm plate. After 5 to 10 days in culture, well-separated colonies were isolated by trypsinization in cloning cylinders and transferred to new 60 mm plates. To induce demethylation, an inactive X-containing hybrid that had never expressed *TIMP1 *(t11-4Aaz-5) was treated with 5-azacytidine as previously described [37]. The cells were grown in media supplemented with HAT (Invitrogen) to select for *HPRT *reactivants and then single cell cloned to test for *TIMP1*-positive cultures. To remove any confounding effects of *HPRT *selection, the cells were transferred back to alpha minimal essential media after 2 weeks of selection. The expression of genes in the *TIMP1 *region was determined by RT-PCR as described previously [12].

### Replication timing

Approximately 4 × 10^6 ^lymphoblast cells were harvested one day after subculture to ensure that they were actively growing. The cell pellet was resuspended in 8 ml of prewarmed hypotonic (0.75 M KCl) and then incubated at 37C for 10 minutes. 2 to 3 ml of fixative (3:1 methanol:glacial acetic acid) was slowly added before spinning at 200 g for 10 minutes. The cell pellet was washed three more times with 5 ml fixative and then resuspended in 10 ml fixative for storage at -20C for up to a week. The nuclei preparations were dropped onto slides and left at room temperature overnight. The slides were then incubated in 2X SSC at 37C for 30 minutes followed by 2 minute room temperature incubations in each of: 70%, 85%, and 95% ethanol, and then allowed to air dry. To denature, the slides were incubated in fresh 70% formamide/2 × SSC at 74C for 2 minutes followed by an ice cold ethanol series of rinses (70%, 85%, 95% for 2 minutes each) and air drying. Probes for both the *TIMP1 *locus (lambda phage TIMP-3.9X) and the *HPRT *locus (Hulambda4x-8, ATCC 57236) were labelled with dUTP-digoxigenin (Roche 1745816) by nick translation (Roche 0976776). Unincorporated nucleotides were removed with a PCR clean-up kit (Qiagen), and 1 ul (approximately 100 ng) of labelled probe was mixed with 10 ul of 70% formamide hybridization buffer and 40 ng of human Cot-1 DNA (Invitrogen 15279011). The probes were denatured at 74C for 10 minutes, pre-annealed at 37C for one hour and then added to the prepared slide to hybridize overnight in a humidified chamber at 37C. The slides were washed in 50% formamide/2 × SSC for 15 minutes at 43C, followed by two washes in 2 × SSC for 4 minutes at 37C and then 3 washes at room temperature for 2 minutes each in 1 × PBD (0.1 M NaH_2_PO_4_, 0.1 M Na_2_HPO_4_, 0.1% Triton X). To visualize the probe, slides were incubated with 500 ng of anti-DIG (sheep – Roche 1207741) conjugated to fluorescein, for 5 minutes at 37C, followed by three two-minute washes in 1 × PBD. To amplify the signal, FITC-anti-sheep (IgG FI-6000 (Vector Laboratories)) was incubated and washed as above. The slides were then counterstained with DAPI mixed with antifade (VectorLabs). Cells were scored for nuclei with single-single, single-double, or double-double *TIMP1 *or *HPRT *signals on a Ziess Axioplan II microscope by a single individual who was blinded as to the cell line being analysed.

### Methylation analyses

For methylation-sensitive restriction enzyme analysis, genomic DNA was pre-digested with *Eco*RI at 37C overnight, followed by incubation with 2 ul of RNase at 37C for 15 minutes. After phenol extraction and ethanol precipitation, the DNA was quantified by spectrophotometry. Pre-digested DNA (2 ug) was then incubated overnight at 37C in a total volume of 20 ul with 20 units of one of the following: mock enzyme (uncut), *Hpa*II, or *Hha*I. An aliquot of 1 ul (100 ng) was then used as a template in the PCR reactions as described previously [14]. All primers flanked a region of genomic DNA that did not contain *Eco*RI restriction enzyme sites and contained 1–2 *Hpa*II or *Hha*I sites. For bisulfite analysis, 500 ng of genomic DNA was first denatured with 3 ul of 3 M NaOH at 37C for 10 minutes. After 15 ul of 20 mM hydroquinone and 255 ul of 3.9 M sodium bisulfite were added and mixed well, reactions were left at 50C for 16 hours to allow the unmethylated cytosines to convert to uracil. DNA was purified using the DNA Wizard Clean-Up Kit (Promega). After amplification with the TIMP-S primers (see Table 3), the 3' reverse oligonucleotide was used as the primer for sequencing of the PCR product.

**Table 3:** Primers for PCR analyses

Primer pair	Sequence	Use
*TIMP1 *5' (promoter) [14]	5'A: CCCTTGGGTTCTGCACTGA* 5'B: CCAAGCTGAGTAGACAGGC	Methylation ChIP DNase sensitivity
*TIMP1 *CA (gene body) [12]	CA1: GGGTTCCAAGCCTTAGGGGA CA2: AGGCTGTTCCAGGGAGCCGC	DNase sensitivity
*TIMP1 *S (bisulfite)	5S: GttttTTGGtTTtTGtAtTGATGGT 3S: CCAAaCTaAaTAaACAaaCATCTAaC**	Bisulfite sequencing
*ARAF1 *M1:M4 (promoter) [14]	M1: TGCCAAAGCCCTAAGGTCA M4: CGCTGTCGACGATGGTCT M3: GTGAGGAAACAAGAAGAGAG	Methylation ChIP DNase sensitivity
*XIST *3':5' (gene body) [39]	3':GAAGTCTCAAGGCTTGAGTTAGAAG 5': TTGGGTCCTCTATCCATCTAGGTAG	Methylation DNase sensitivity
*XIST *A5:29r (promoter) [37]	A5: TTTCTTACTCTCTCGGGGCT 29r: ATCAGCAGGTATCCGATACC	ChIP
*ELK1 *5' (promoter) [14]	A: GCACAGCTCTGTAGGGAA B: AGCTCACCTGTGTGTAGCG	Methylation ChIP
STA A:B (intergenic)	A: CACCTGTGTGTCATGTATAC B: CCAGTATTGGTCTTCCAGTT	DNase sensitivity
8037 A:B (intergenic)	A: GAGGCAAGACATCCATTCC B: TGACTTTGAGCGAGCAGGT	Reference region

* There is a mismatch in the TIMP 5'A primer, the underlined G should be C.

** The lower-case letters in the primers are the bases modified by the bisulfite reaction. All C nucleotides should have been converted because there were no CpG pairs with possible protective methylation.

### RNase Protection quantitation of *TIMP1 *levels

RNase protection analysis was performed with Ambion's RPA II kit, following the manufacturer's directions. RNA probes were isolated after *in vitro *transcription with ^32^P-UTP. After solution hybridization overnight of excess antisense radiolabelled probe to 10 ug of total RNA, any unhybridized probe and sample RNA was removed by RNase digestion. The hybridized product was then separated on a native 5% polyacrylamide gel, visualized by autoradiography, and bands quantified by phosphoimager (BioRad FX). The intensity of the *TIMP1 *fragment was compared to the intensity of the band detected for *MIC2 *used to control for the amount of input RNA. All RPA results were normalized to one stock RNA to decrease variability between gels (see [14]).

### DNaseI sensitivity

The protocol for nuclease sensitivity was adapted primarily from [38]. The somatic cell hybrids were grown to 75% confluence on 60 mm plates before harvesting with 0.25% trypsin-EDTA. The cell pellets were resuspended in 300 ul ice-cold DNase buffer (0.3 M sucrose, 60 mM KCl, 15 mM NaCl, 5 mM MgCl_2_, 0.1 mM EGTA, 0.5 mM DTT, and 15 mM Tris-HCL pH7.5). The suspensions were then split into 6 tubes of 50 ul each and another 50 ul of ice-cold DNase buffer with 0.4% Nonidet P40 was added. The tubes were mixed gently and placed on ice for four to five minutes before 100 ul of freshly diluted DNaseI (Invitrogen 18047-019) was added, such that 2.5 U, 1 U, 0.5 U, 0.25 U, 0.1 U, and no enzyme were used. The reaction was then incubated at 25C for 5 minutes, followed by 95C for 15 minutes. The DNA was isolated with a standard phenol/chloroform extraction protocol, quantified with spectrophotometry, and diluted to 60 ng/ul, 12 ng/ul, and 4 ng/ul. 1 ul was used as a PCR template with primers listed in Table 3. The diluted DNAs showed product intensity inversely proportional to concentration, indicating that the PCRs at 25 cycles of amplification were in the linear range of amplification, and the results shown in figure 3 are for the 12 ng/ul template.

### Chromatin Immunopreciptation Assay (ChIP Assay)

The hybrid cells were grown in a t25 flask and were harvested after treatment with 5 drops of 0.25% trypsin-EDTA for 2 minutes at room temperature, and then washed in 1 × PBS. The cells were placed in a 1.5 ml tube with one ml of 0.37% formaldehyde in minimal essential media (Invitrogen) and incubated at 37C for ten minutes. From this point, the cells were kept on ice. The cells were washed twice with 1/100 proteinase inhibitor cocktail (Sigma) in 1 × PBS and then centrifuged at 2500 rpm for 4 minutes at 4C. The pellet was resuspended in 200 ul SDS lysis buffer (Upstate) with 1/100 proteinase inhibitor cocktail and placed on ice for 10 minutes. The suspension was drawn up with a 25 gauge needle and then sonicated. Immunoprecipitation was performed (Upstate Biochemicals catalogue number 17-295) with the following antibodies: Anti-acetyl H3 against lysine 9 and 14 (catalogue number 06-599); Anti-acetyl H4 against lysine 5, 8, 12, and 16 (catalogue number 06-866); and Anti-dimethyl-H3 against lysine 9 (catalogue number 07-212).

## Abbreviations

H3 – histone H3; H4 – histone H4; K – lysine; ChIP – chromatin immunoprecipitation; X – X chromosome; Y – Y chromosome; Xi – inactive X; Xa – active X; M – methylated; U – unmethylated; + expressing; - not expressing; S – single signal; D – double signal.

## Authors' contributions

CA carried out the molecular genetic studies and data analysis and drafted the manuscript. CB conceived of the study, participated in the experimental design and data analysis and wrote the final manuscript. Both authors read and approved the final manuscript.
